# Characterization of Solid-State Drug Polymorphs and Real-Time Evaluation of Crystallization Process Consistency by Near-Infrared Spectroscopy

**DOI:** 10.3389/fchem.2018.00506

**Published:** 2018-10-22

**Authors:** Shu-Ye Qi, Ye Tian, Wen-Bo Zou, Chang-Qin Hu

**Affiliations:** ^1^National Institutes for Food and Drug Control, Beijing, China; ^2^State Key Laboratory of Biochemical Engineering, Institute of Process Engineering, Chinese Academy of Sciences, Beijing, China

**Keywords:** process consistency, drug polymorph, solid-state nuclear magnetic resonance spectroscopy, near-infrared spectroscopy, Raman microscopy

## Abstract

Herein, we aimed to develop a strategy for evaluating the consistency of pharmaceutically important crystallization processes in real time, focusing on two typical cases of polymorphism. Theoretical analysis using a combination of ^13^C solid-state nuclear magnetic resonance spectroscopy with other polymorphism analysis techniques identified a number of marker signals, the changes of which revealed the presence of two or more structural orientations (lattices and/or molecular conformations) in both cefazolin sodium pentahydrate (α-CEZ-Na) and cephathiamidine (CETD). The proportions of these forms were shown to be batch-dependent and were defined as critical quality attributes (CQAs) to evaluate process consistency. Subsequently, real-time analysis by chemometrics-assisted near-infrared spectroscopy (NIR) was used to obtain useful information corresponding to CQAs. The pretreated spectra of representative samples were transformed by first derivative and vector normalization methods and used to calculate standard deviations at each wavelength and thus detect significant differences. As a result, vibrational responses of H_2_O, CH_3_, and CH_2_ moieties (at 5,280, 4,431, and 4,339 cm^−1^, respectively) were shown to be sensitive to the CQAs of α-CEZ-Na, which allowed us to establish a highly accurate discrimination model. Moreover, signals of H_2_O, CONH, and COOH moieties (at 5,211, 5,284, and 5,369 cm^−1^, respectively) played the same role in the case of CETD, as confirmed by theoretical results. Thus, we established a technique for the rapid evaluation of crystallization process consistency and deepened our understanding of crystallization behavior by using NIR in combination with polymorphism analysis techniques.

## Introduction

Polymorphism is defined as the ability of a solid substance to exist in various crystal forms (i.e., polymorphs) that exhibit different physical properties such as density, melting point, solubility, and dissolution rate. Therefore, the interconversion of the polymorphic forms of a given drug can strongly affect its bioavailability and therapeutic effect (Aaltonen et al., 2009), which makes crystallization process control a matter of critical importance for the pharmaceutical industry. According to the quality by design (QbD) approach, the quality target product profile (QTPP) is mostly described by critical quality attributes (CQAs), a deep understanding of which is required for the identification of critical material attributes (CMAs) and critical process parameters (CPPs) in the production process. In turn, the establishment of a relationship between CMAs, CPPs, and CQAs enables the quality control of crystallized products (Yu, 2008; Yu et al., 2014). Thus, the identification of CQAs for polymorphic drugs is a key part of their development and production.

Solid-state substances can be characterized by analytical techniques such as powder X-ray diffraction (PXRD), IR spectroscopy, Raman spectroscopy, and solid-state nuclear magnetic resonance spectroscopy (ssNMR) (Shah et al., 2006). Among them, PXRD is most useful for the identification of crystal forms but requires known or constant crystallinity for analysis of polymorph mixtures. IR spectroscopy exhibits the advantage of easy sample preparation; however, the application of pressure during sample preparation/manipulation (e.g., for ATR and KBr disk techniques) may induce solid-state phase changes (Calvo et al., 2017). Raman spectroscopy exhibits suitable sensitivity toward polymorphism, hydrate formation, and crystallinity but is a surface technique, which implies that particle size and surface homogeneity control are needed for more accurate and reproducible results (Hédoux et al., 2011). To some extent, the above defects can be mitigated by employing Raman microscopy. Finally, ssNMR suffers from insensitivity to particle size (Brown and Spiess, 2001) but provides rich structural information because of its exquisite sensitivity toward minor conformational changes and has therefore been widely used to study pharmaceutical polymorphism (Harris, 2007). The above methods are fully mutually orthogonal, and the strengths and weaknesses of each technique have to be exploited on a case-by-case basis to reliably develop a suitable characterization methodology. In this study, we combine ssNMR with PXRD and Raman microscopy to characterize the differences (defined as CQAs) between solid-state forms of drugs from various batches.

Since the 1990s, near-infrared spectroscopy (NIR) has steadily evolved to become a popular method of quantifying pharmaceutically relevant materials in the solid state. This technique exhibits numerous advantages that make it well suited for real-time quality monitoring, e.g., it is fast, non-destructive, and does not require sample preparation (Bakeev, 2010; Karry, 2014). However, NIR signals appear as abundant broad overlapping bands, which require the utilization of chemometrics to remove interferences, extract useful information, and establish qualitative or quantitative models. Herein, we employed a set of data processing methods to develop a technique for real-time crystallization process monitoring by NIR.

Two drugs were chosen as typical cases to develop and test the above technique, namely cefazolin sodium pentahydrate (α-CEZ-Na), which exhibits a well-defined and relatively fixed solid-state form without isomorphic dehydration, and cephathiamidine (CETD), the structural orientations of which are hard to determine and are strongly affected by manufacturing conditions (Kamat et al., 1988; Mimura et al., 2002; Hao et al., 2017).

## Materials and methods

### Materials

The 150 samples of α-CEZ-Na from two processing periods (Period 1: July 2014–January 2015 and September 2015; Period 2: November 2016–March 2017) were provided by Shenzhen Gosun Pharmaceutical Co., Ltd., Guangdong, China (Gosun). To amplify the differences between products, α-CEZ-Na 1–3 (batch no: 1203283, 1203423, 1203403) from Gosun, α-CEZ-Na 4 (batch no: L100200) from Fujisawa Co., Ltd., Kirihara-cho, Fujisawa-shi, Kanagawa, Japan (Fujisawa), and α-CEZ-Na 5 (batch no: 1391) from Kyongbo Phar. Co., Ltd., Silok-ro, Ahsan-si, Choongchungnam-do, South Korea (Kyongbo) were chosen as representative samples for comparison of theoretical characteristics. The quality of every α-CEZ-Na sample complied with the Japanese Pharmacopeia.

The 96 samples of CETD from different batches were provided by Guangzhou Baiyunshan Pharmaceutical Co., Ltd., Guangzhou, China. Among them, CETD 1 (batch no: 1611017), CETD 2 (batch no: 1612010), CETD 3 (batch no: 1703001), CETD 4 (batch no: 1701008), and CETD 5 (batch no: 1704016) were chosen as representative samples to analyze theoretical characteristics. The quality of every CETD sample complied with the Chinese Pharmacopeia.

### SsNMR

All ssNMR spectra were recorded on a Bruker AVANCE II WB400 NMR spectrometer using scanning parameters referenced elsewhere (Tian et al., 2018).

### Raman

Raman mapping was performed using a DXRxi Raman imaging microscope (Thermo Fisher Scientific Inc., Hudson, USA) equipped with a 532 nm excitation laser. The laser power, exposure time, number of scans, and image pixel size equaled 6.0 mW, 0.00286 s, 11, and 5.0 μm, respectively. System control and spectrum acquisition were conducted using the ThermoScientific OMNIC software.

### NIR

Samples were directly scanned in vials using a Fourier transform NIR integrating sphere (MPA, Bruker, Switzerland). The scan wavelength range and resolution were set to 12,000–4,000 cm^−1^ and 8 cm^−1^, respectively. All spectra were obtained by averaging the results of 32 scans, and six spectra of the same sample were averaged to a representative mean spectrum.

### Data handling

Raman mapping produced microscopic images as three-dimensional data sets that comprised two-dimensional data matrices containing information on the number of pixels and the number of data points in each spectrum. Before the application of any imaging algorithm, each individual spectrum (3,400–50 cm^−1^) was compared with all other spectra within a given data set. The largest variations were observed for the relative intensities at 1,644 and 1,658 cm^−1^, and the ratio of these intensities (*I*_1644_/*I*_1658_) was used as the third dimension for image mapping.

Chemometrics is widely used to transform original NIR spectra and establish qualitative or quantitative models. Herein, first derivative (1st Der) transformations using the Savitzky-Golay (SG) convolution filter (17, 3) and vector normalization (Chu, 2011) were chosen to improve NIR performance, and multiple linear regression for discriminant analysis (MLR-DA) or hierarchical cluster analysis (HCA) (Hedegaard et al., 2011) were employed to realize on-line process evaluation.

## Results and discussion

### Case 1: α-CEZ-Na

#### Theoretical analysis of CQAs for α-CEZ-Na

Our previous study (Tian et al., 2018) showed that differences between α-CEZ-Na samples sourced from three vendors (Gosun, Kyongbo, and Fujisawa) could be directly observed by PXRD; however, the above technique did not allow the detection of subtle variations, which was mitigated by the use of ssNMR.

**Figure 1:**
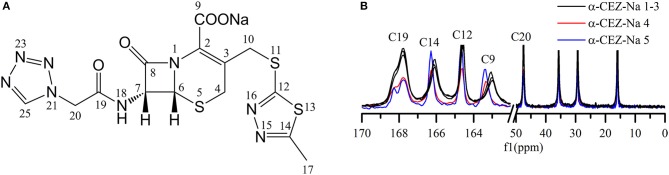
**(A)** Structure of cefazolin sodium showing the numbering of the carbons; **(B)** ^13^C ssNMR spectra of α-CEZ-Na 1–5 produced by different vendors (Gosun, Fujisawa, and Kyongbo).

Distinct differences were observed for the shape of peak C19 (i.e., for the intensity of the shoulder peak of C19) and the chemical shifts of peaks C14 and C9 (Figures 1A,B). Based on the computed conformations of CEZ-Na, the shoulder peak of C19 observed in ^13^C ssNMR spectra was assigned to the formation of intramolecular hydrogen bonds between N22 and N18 in conformation 2 (Tian et al., 2018). Comparison of the relative intensity of this shoulder peak for α-CEZ-Na 1–5 revealed that the fractions of conformation 2 in Gosun samples were significantly lower than those in Kyongbo samples, but close to those in Fujisawa samples. The chemical shift differences of C14 between different polymorphs equaled ~0.1 ppm, which was probably ascribed to the corresponding variation of van der Waals forces, whereas a difference of 0.4 ppm observed for the chemical shift of C9 may be attributed to changes of electrostatic interactions between sodium ion and the COO^−^ residue of C9, as well as intermolecular hydrogen bonds between crystal water and the COO^−^ residue of C9. All of these differences were influenced by the proportions of different conformations in the product and could be defined as the CQAs of α-CEZ-Na to assess the variability of the crystallization process. The spectra of α-CEZ-Na 1–3 revealed that Gosun samples featured similar ssNMR signals; however, the above CQAs were still suitable for investigating process consistency.

#### NIR analysis of CQAs for α-CEZ-Na

**Figure 2:**
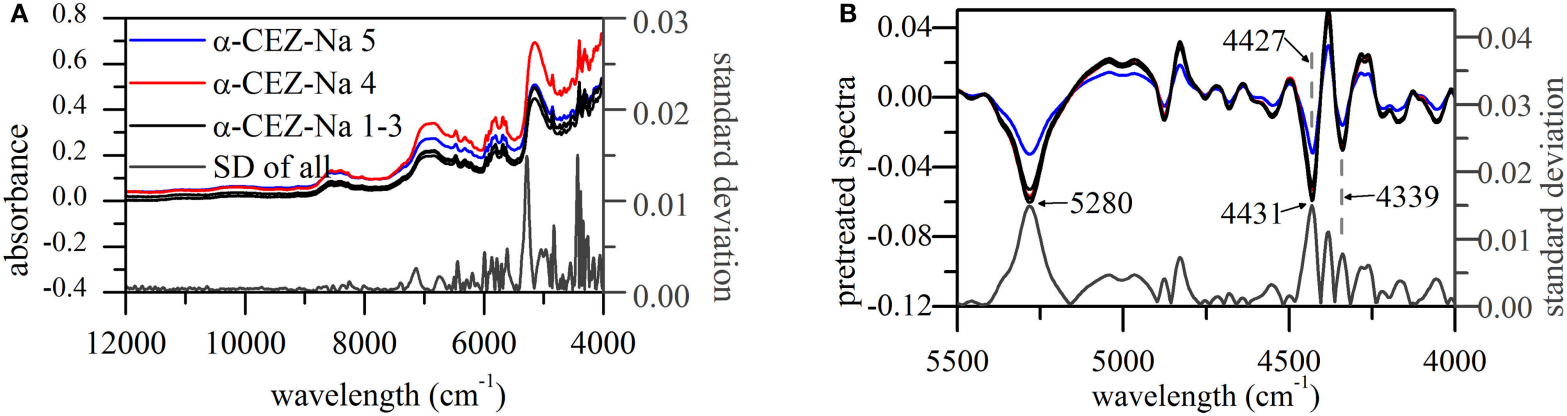
NIR spectra of α-CEZ-Na 1–5 produced by different vendors (Gosun, Fujisawa, and Kyongbo). **(A)** Their original spectra from 4,000~12,000 cm^−1^ and SDs of pretreated spectra at each wavelength; **(B)** pretreated spectra and their SDs from 4,000~5,500 cm^−1^, and the pretreated spectra were the ones transformed by first deviation and vector normalization.

Standard deviations (SDs) of pretreated spectra were calculated to reveal the dispersion of response values at a given wavelength (Figure 2A). As a result, notable differences were observed in the range of 5,500–4,000 cm^−1^, especially around 5,280, 4,431, 4,381, and 4,339 cm^−1^. The signals at 5,280, 4,431, and 4,339 cm^−1^ could return to their original positions around 5,164, 4,405, and 4,335 cm^−1^, respectively; however, this behavior was not observed for the signal at 4,381 cm^−1^. According to a previous study (Li, 2006; Lu, 2007), absorbances at 5,164, 4,405, and 4,335 cm^−1^ can be used to extract information on O–H stretching (str.) and O–H deformation (def.) vibrations of H_2_O and C–H str./def. vibrations of CH_3_ and CH_2_ moieties, respectively.

CEZ-Na contained only one methyl group (C17), the vibration of which was probably described by the absorption band at 4,405 cm^−1^. The above methyl group was connected to C14 and was therefore expected to feature a vibration performance dependent on the variation of C14 electron cloud density, which was likely to happen according to the results of ssNMR analysis. Figure 2B shows that the peak at 4,431 cm^−1^ shifted to 4,427 cm^−1^ only for α-CEZ-Na 5, i.e., for the sample characterized by a significantly high proportion of conformation 2. On the other hand, substituent folding at C7 was expected to induce a vibrational change of C20 CH_2_, and another CH_2_ group (C4) might be also affected by the concomitant space narrowing. However, the vibration mode of C10 CH_2_ was expected to be unaffected and was concluded to correspond to the peak at 4,339 cm^−1^ that retained its position in all cases.

**Figure 3:**
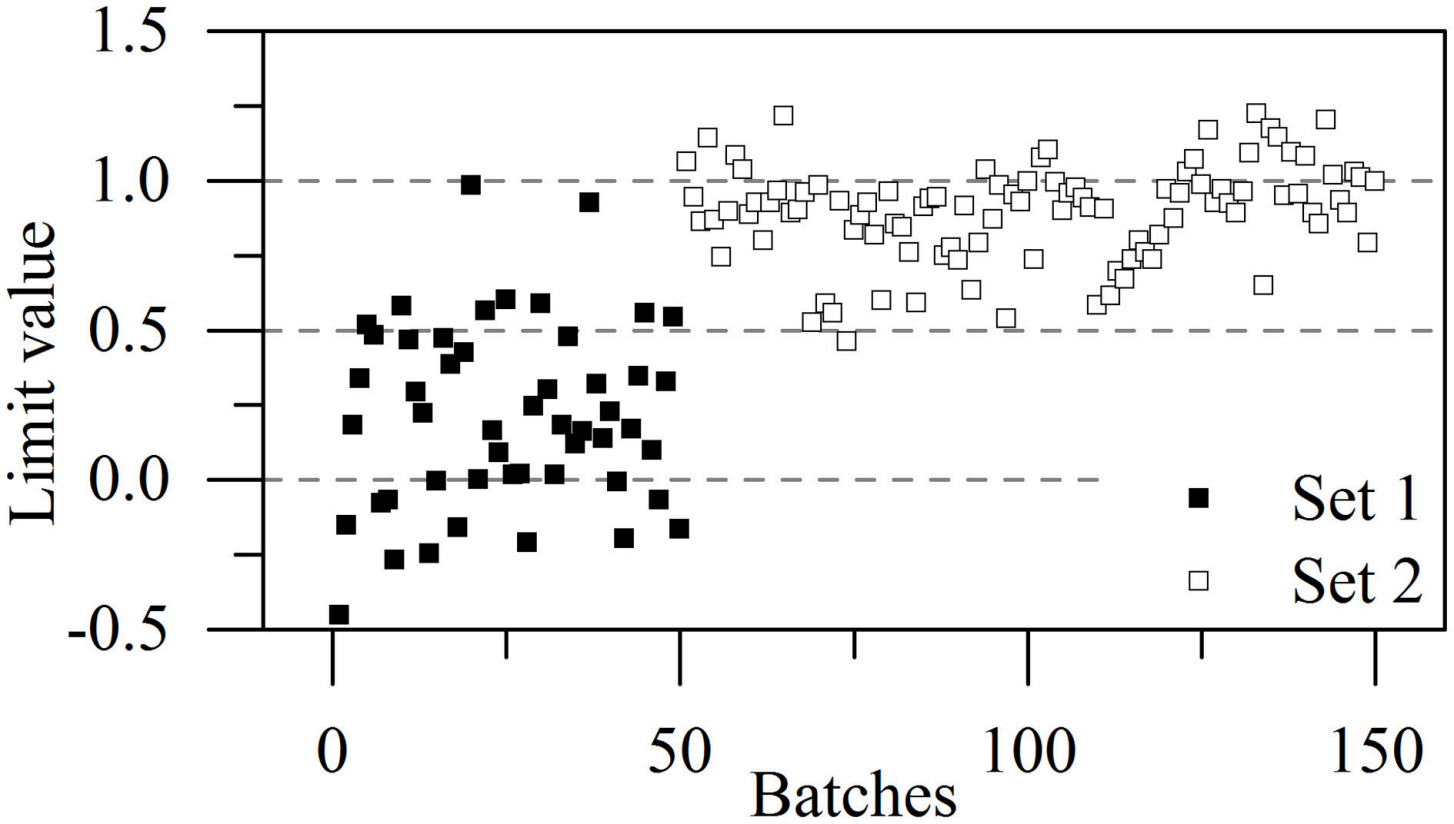
MLR-DA results for all α-CEZ-Na products obtained in Periods 1 and 2.

The higher proportion of conformation 2 in Kyongbo samples with lower NIR intensities (Figure 2B) indicated that folding arrangements differing in the pattern of intramolecular hydrogen bonds weakened the interaction between specific groups and NIR photons. Herein, the intensities observed for pretreated spectra at wavenumbers of 5,280, 4,431, and 4,339 cm^−1^ were regarded as three variables (*x*_1_, *x*_2_, and *x*_3_) to establish an MRL-DA model. To highlight crystallization process differences for the same vendor (Gosun), α-CEZ-Na samples of different processing periods were chosen for consistency analysis. Fifty samples from Period 1 were defined as Set 1 (limiting value: *y*_1_ = 0 ± 0.5), and 100 samples from Period 2 were defined as Set 2 (limiting value: *y*_2_ = 1 ± 0.5). Figure 3 shows that the above method achieved a 94% classification accuracy for the tested samples. Comparison of predicted value SDs (SD_1_ = 0.31, SD_2_ = 0.16) indicated that the Gosun crystallization process was modified and that its consistency improved upon going from Period 1 to Period 2. The fact that lower NIR intensities were observed for Period 2 than for Period 1 implied that the proportion of conformation 2 increased in α-CEZ-Na upon going from Period 1 to Period 2. Therefore, it would be excellent if the folded structure was needed to improve the efficacy of α-CEZ-Na. Taking into account the physical property differences between various solid-state forms, the proportion of discrepant conformations should be strictly controlled in view of safety considerations, which could be achieved by adopting the above NIR strategy to evaluate the consistency of the crystallization process and monitor the corresponding changes on-line.

### Case 2: CETD

#### Theoretical analysis of CQAs for CETD

**Figure 4:**
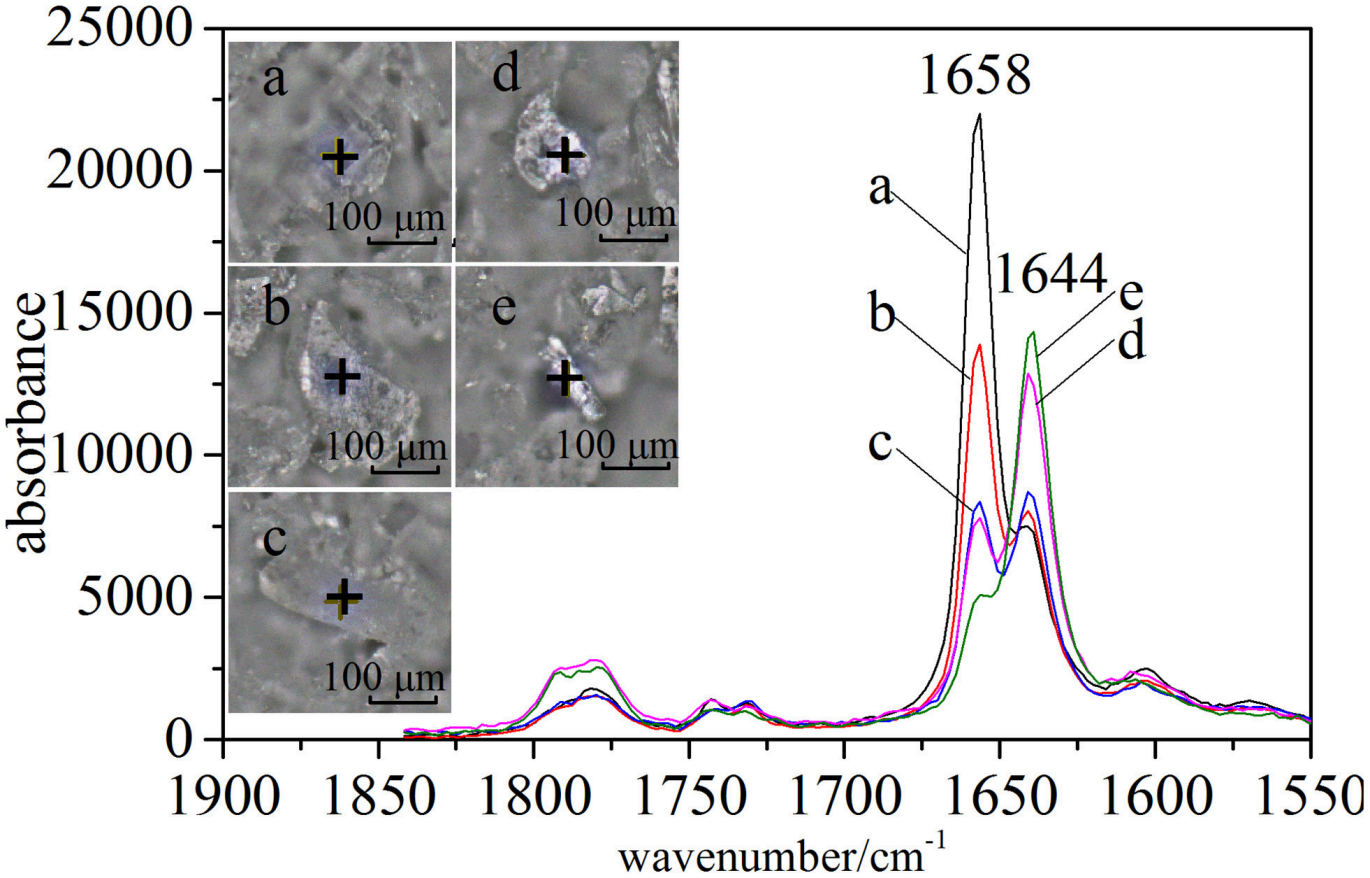
Raman spectra (1,550–1,830 cm^−1^) of five CETD crystals marked in optical images by “+”.

In the case of CETD, the Raman spectra of specified positions were scanned to reflect the differences of solid-state forms (Figure 4). Under a microscope, CETD samples appeared as a combination of plate-like and rod-like crystals that could be distinguished based on the relative intensities of peaks at 1,658 and 1,644 cm^−1^. For plate-like crystals, the peak at 1,658 cm^−1^ was always more intense than that at 1,644 cm^−1^, whereas the opposite was true for rod-like crystals. The absorption peaks around 1,658 and 1,644 cm^−1^ were ascribed to the vibrations of free and associated amide C = O moieties at C15, respectively, and the lower absorption frequency and larger width of the latter peak were ascribed to the formation of hydrogen bonds. The above findings indicated the presence of at least two crystal types in CETD. However, Raman microscopy simply provided local information on polymorphic CETD.

The ^13^C NMR data obtained in dimethyl sulfoxide-*d*_6_ (DMSO-*d*_6_) were typically similar to those extracted from ssNMR spectra. Hence, the data assigned based on ^13^C NMR results served as guide for the assignment of ^13^C ssNMR data. Specifically, the ^13^C ssNMR signals of CETD were accurately assigned using the techniques of distortionless enhancement of polarization transfer, ^1^H-^1^H homonuclear correlation spectroscopy, heteronuclear single quantum coherence, and heteronuclear multiple bond correlation (Table 1).

**Table 1:** NMR data (δ) for CETD.

**Assignment**	**^13^C NMR in DMSO-*d_6_***	**ssNMR**
C2	115.8	114.4; 106.9
C3	131.9	132.8; 131.3
C4	25.3	24.2; 23.3
C6	56.8	58.4
C7	58.9	53.9; 53.3
C8	162.8	158.7
C9	163.6	162.8; 161.5
C10	63.8	69.0; 63.1
C12	170.4	170.1
C13	20.7	19.3^a^
C15	170.1	169.1
C16	34.0	32.8; 29.8
C18	170.1	167.5; 165.7
C20	45.5	44.1; 43.4
C21(C21′)	22.9	19.3^a^
C23	49.3	48.1
C24(C24')	22.9	19.3^a^

a *overlapped peaks*.

**Figure 5:**
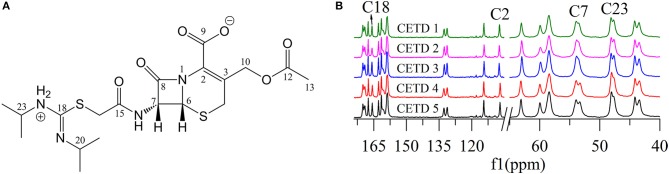
**(A)** Structure of cephathiamidine showing the numbering of the carbons; **(B)** ^13^C ssNMR spectra of CETD 1–5 obtained from different batches.

Figure 5 shows that two distinguishable ssNMR peaks were observed for a number of CETD carbon sites, which implied the coexistence of two molecules in the asymmetric unit, i.e., chemical shift differences were ascribable to the asymmetric crystal packing in the solid (Wang et al., 2012). Comparison of the spectra of CETD 1–5 revealed distinct differences in the shape of C7 and C23 peaks (i.e., in the intensities of the corresponding shoulder peaks) and in the chemical shift of peaks C2 and C18. Notably, the spectra of CETD 1, 2, and 3 were similar and exhibited the following common features (1) the chemical shifts of C2 and C18 equaled 107.3 and 161.1 ppm, respectively; (2) the shoulder peak of C7 was weak or even non-existent; and (3) the shoulder peak of C23 was clearly visible. Conversely, the shoulder peak could be obviously observed for peak C7 but not for peak C23 in the cases of CETD 4 and 5, which demonstrated the possible existence of hydrogen bonds separately connecting the NH${}_{2}^{+}$ residue of N22 or the CONH residue of C15 in different conformations. Furthermore, the C2 and C18 chemical shift differences between CETD 1–3 and CETD 4–5 both equaled ~0.4 ppm (Δδ = 107.3–106.9 = 0.4 ppm and Δδ = 166.1–165.7 = 0.4 ppm, respectively), which suggested that these changes might reflect variations of electrostatic interactions or hydrogen bonding connecting the COO^−^ residue of C9 and the NH${}_{2}^{+}$ residue of N22. The above findings indicated the presence of two or more different structural orientations (lattice structures and/or molecular conformations) in the studied CETD samples, the proportions of which varied between batches. Thus, these theoretical results identified subtle variations in different batches of CETD and demonstrated that these variations can be regarded as CQAs of the corresponding crystallization process.

#### NIR analysis of CQAs for CETD

**Figure 6:**
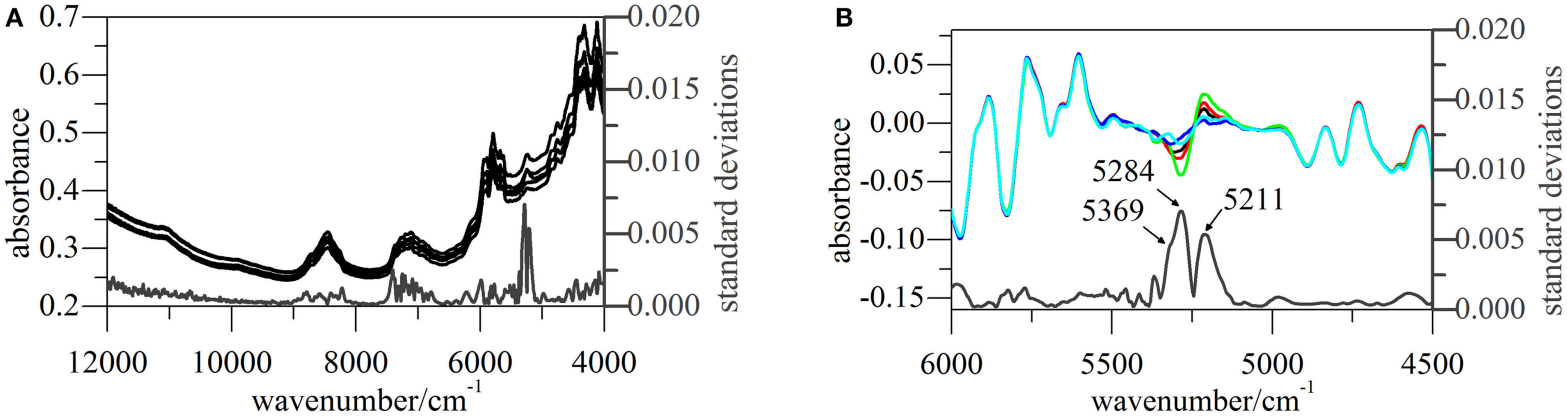
NIR spectra of CETD 1–5 obtained from different batches. **(A)** Their original spectra from 4,000~12,000 cm^−1^ and SDs of pretreated spectra at each wavelength; **(B)** pretreated spectra and their SDs from 4,500~6,000 cm^−1^, and the pretreated spectra were the ones transformed by first deviation and vector normalization.

All NIR spectra of CETD were transformed by SG(17, 3) 1st Der and vector normalization, and SDs at each wavelength were calculated for the intensities of these pretreated spectra (Figure 6). The largest variations were observed for signals at 5,211, 5,284, and 5,369 cm^−1^, which indicated the need to scrutinize peaks located at 5,168, 5,238, and 5,273 cm^−1^ in original spectra (O–H str. and O–H def. of H_2_O, C = O str. second overtone of CONH, and C = O str. second overtone of COOH, respectively) (Li, 2006; Lu, 2007). According to the molecular structure of CETD, the transformed intensities at 5,284 and 5,369 cm^−1^ could be assigned to the vibration of CONH at C15 and COOH at C9, and the above results were therefore in good agreement with those of theoretical CQAs analyses.

**Figure 7:**
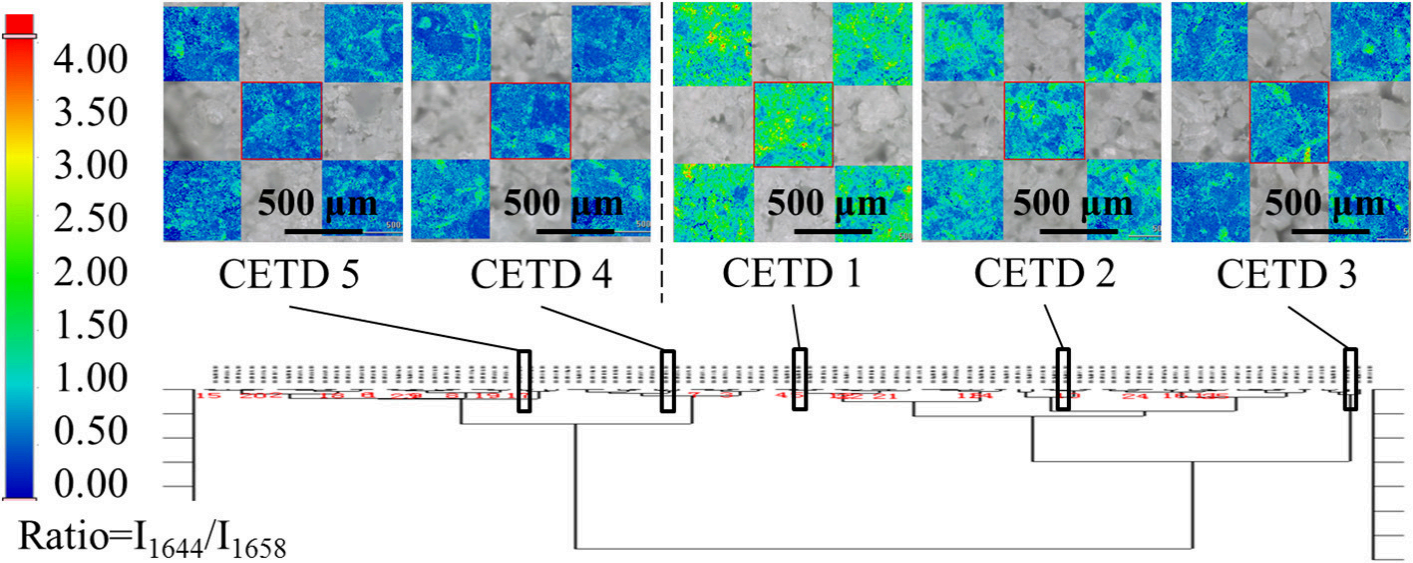
Results of HCA (Ward's method) performed using intensities of peaks at 5,211, 5,284, and 5,369 cm^−1^ in pretreated NIR spectra of all samples. Among these results, CETD 1–5 were emphasized by their own microscope Raman images, which mapping by the ratio of intensities at 1,644 and 1,658 cm^−1^ (*I*_1644_*/I*_1658_).

All samples were classified according to the intensities of peaks at 5,211, 5,284, and 5,369 cm^−1^ in pretreated spectra using HCA. Based on the results in Figure 7, 96 samples were well divided into two classes, namely CETD 1–3 and CETD 4–5, in agreement with the results of ssNMR spectroscopy. Microscopy-coupled Raman imaging revealed that CETD 1–3 had a higher proportion of rod-like crystals than CETD 4–5, as reflected by the higher *I*_1644_/*I*_1658_ > 1.00 value of the former. Thus, NIR was proven to be a suitable technique for determining the CQAs of CETD.

## Discussion

**Chart 1:**
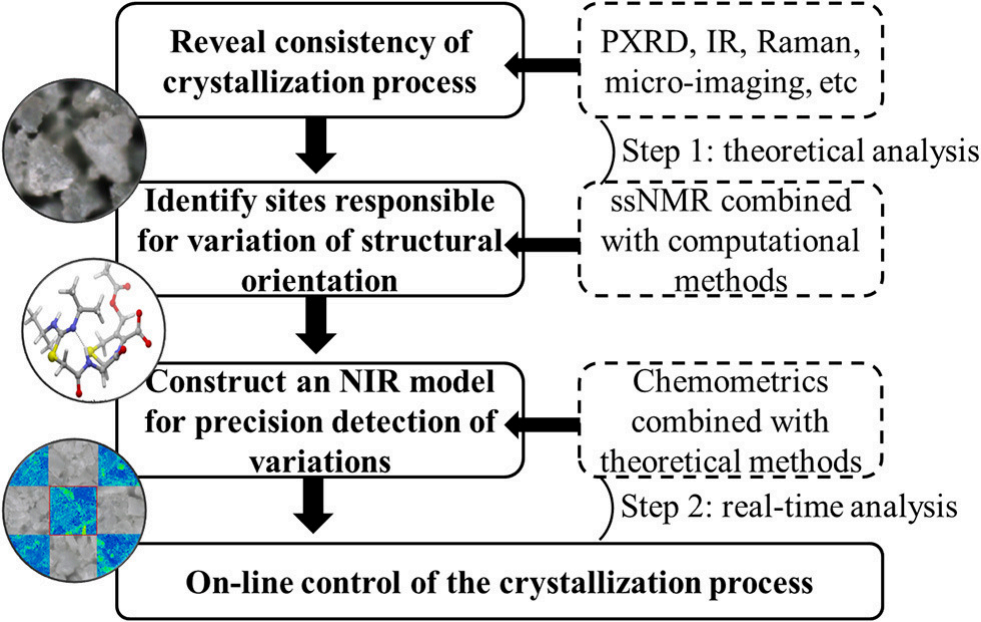
Strategy to control of critical quality attributes of polymorphic drugs in crystallization process.

ssNMR and Raman microscopy allowed for effective characterization of the mixed solid-state forms of investigated drugs, and changes of peak shape and/or peak intensity were shown to be sensitive to those of polymorph proportions. These variations could be defined as CQAs for each drug to reveal the consistency of the corresponding crystallization processes. Considering the requirement of real-time detection, NIR was identified as a practical technology to replace theoretical analysis methods despite requiring the assistance of chemometrics. First derivative and vector normalization were proposed as methods of transforming NIR spectra to achieve baseline correction and high resolution, and SD spectra of representative samples allowed one to easily find variables corresponding to CQAs. Therefore, these characteristic features could be used to establish a model for the rapid on-line evaluation of API crystallization degree or the consistency of mixed polymorphs. Most importantly, this strategy (Chart 1) enabled the continuous monitoring of crystallization process changes to achieve the desired objective of drug efficacy and safety.

However, revealing the reason of process variation scientifically remains a challenge even for modern analytical techniques, necessitating the use of supporting methods such as terahertz, IR and thermogravimetric analysis in certain cases. In addition, computational chemistry methods are required for clarifying certain structural aspects in detail. For seeking right protective measures of process consistency, NIR analysis can be assisted by other chemometric algorithms as long as they benefit the extraction of key information corresponding to CQAs.

## Author contributions

S-YQ: design of experiments, Raman and NIR data acquisition, analysis of experimental data; YT: SSNMR data acquisition; W-BZ: PXRD data acquisition; C-QH: design of experiments, analysis of experimental data.

### Conflict of interest statement

The authors declare that the research was conducted in the absence of any commercial or financial relationships that could be construed as a potential conflict of interest.
